# MicroRNA-138 functions as a tumor suppressor in osteosarcoma by targeting differentiated embryonic chondrocyte gene 2

**DOI:** 10.1186/s13046-016-0348-5

**Published:** 2016-04-19

**Authors:** Baoen Jiang, Weidong Mu, Jiangquan Wang, Jianshu Lu, Shanyong Jiang, Liang Li, Haining Xu, Hongyan Tian

**Affiliations:** Department of Traumatic Orthopaedics, The People ’s Hospital of Dongying City of Shandong Province, No 317 Nanyi Road, Dongying, 257091 Shandong China; Department of Traumatic Orthopaedics, Shandong Provincial Hospital Affiliated to Shandong University, No 324 Jingwuweiqi Road, Jinan, 250021 Shandong China

**Keywords:** MicroRNA-138, Osteosarcoma, Differentiated embryonic chondrocyte gene 2, Proliferation, Apoptosis, Invasion

## Abstract

**Background:**

MicroRNA-138 (miR-138) has been proven to be a tumor suppressor gene in various types of tumors. However, the expression and the role of miR-138 in human osteosarcoma are still poorly understood. We investigated the function and the underlying mechanism of miR-138 in osteosarcoma.

**Methods:**

The expression of miR-138 in human osteosarcoma tissues and cell lines was detected by real-time PCR analysis. The gain-of-function and loss-of-function experiments were performed on osteosarcoma cell lines to investigate the effects of miR-138 on osteosarcoma progression, and to determine whether differentiated embryonic chondrocyte gene 2 (DEC2) mediates these effects. Cell proliferation, apoptosis and invasion were assessed by MTT, flow cytometry and transwell-matrigel assays. Dual-luciferase reporter assay was used to identify whether DEC2 is a direct target of miR-138.

**Results:**

MiR-138 was significantly downregulated in human osteosarcoma tissues and cell lines. Moreover, miR-138 expression was significantly lower in metastatic osteosarcoma tissues than that in non-metastatic tissues. The in vitro gain-of-function and loss-of-function experiments demonstrated that miR-138 inhibited cell proliferation and invasion, and promoted cell apoptosis of human osteosarcoma cells. DEC2 was verified as a direct target of miR-138, and DEC2 could reverse the inhibitory effect of miR-138 on osteosarcoma progression.

**Conclusions:**

These findings suggested that miR-138 acts as a tumor suppressor in osteosarcoma.miR-138 inhibited cell proliferation and invasion, as well as promoted cell apoptosis of human osteosarcoma cells, at least partially, by inhibiting the expression of DEC2. MiR-138/DEC2 may be a novel therapeutic target in osteosarcoma.

## Background

Osteosarcoma is the most common primary malignant bone tumor in children and young adults, comprising 2.4 % of all malignancies in pediatric patients, and about 20 % of all primary bone tumors [1]. Osteosarcoma is highly aggressive, and the 5-year event-free survival rate for patients with metastatic osteosarcoma is only 14 % [2]. Therefore, elucidating the molecular mechanisms for osteosarcoma metastasis and exploring molecular markers to predict tumor aggressiveness are urgently needed.

MicroRNAs (miRNAs or miRs) are small non-coding RNAs that control cellular function by negatively modulating gene expression at either post-transcriptional or translational levels [3–5]. In recent years, the role of miRNAs in the pathogenesis of cancers has been extensively studied [6–9]. The deregulation and aberrant expression of miRNAs is well-recognized to contribute to the development of osteosarcoma [10, 11]. MiR-138 is a frequently downregulated miRNA in various types of tumors, including colorectal cancer, head and neck squamous cell carcinoma (HNSCC), cholangiocarcinoma, and lung cancer [12–16]. Several studies have indicated that downregulation of miR-138 promotes the progression of tumorigenesis [12, 14, 17–19]. Poos et al. suggest that miR-138 is related to osteosarcoma cell proliferation [20]. However, the expression of miR-138 and its role in human osteosarcoma are still poorly understood.

Differentiated embryonic chondrocyte gene 2 (DEC2) is a basic helix-loop-helix transcription factor which has been suggested to play key roles in hypoxia response, cellular proliferation, cell cycle and circadian regulation, and carcinogenesis [21–27].

DEC2 has been implicated to act as a tumor suppressor in breast, endometrial, pancreatic and oral cancers [21, 28, 29]. In contrast to these types of cancers, a study by Hu et al. indicated that DEC2 may contribute to the development and progression of osteosarcoma [30].

In the present study, we investigated the expression and biological function of miR-138 in osteosarcoma. We found miR-138 expression was downregulated in human osteosarcoma tissues and cell lines. We provided the in vitro evidence that miR-138 inhibits osteosarcoma cell proliferation and invasion, and promotes osteosarcoma cell apoptosis. Moreover, we demonstrated that DEC2 was a direct target of miR-138. This study provides new insights into the pathogenesis of osteosarcoma, and contributes to developing novel therapeutic strategies for osteosarcoma.

## Methods

### Patients and tissue samples

This study was approved by the Ethics Committee of The People ’s Hospital of Dongying City of Shandong Province. All the patients (or patients’ parents on behalf of the children) signed an informed consent form prior to study enrollment. 65 osteosarcoma specimens and the adjacent normal bone tissues (located > 3 cm away from the tumor) were obtained from 65 osteosarcoma patients who underwent surgery at the The People ’s Hospital of Dongying City of Shandong Province. The clinical characteristics of these patients were shown in Table 1. Fresh tissues were stored in liquid nitrogen before RNA extraction.

**Table 1:** Clinical characteristics of patients with osteosarcoma

Parameter	Cases (%)
Age (years)	
≤ 15	24 (36.9)
> 15	41 (63.1)
Gender	
Male	39 (60.0)
Female	26 (40.0)
Sites	
Femur	44 (67.7)
Tibia	14 (21.5)
Humerus	4 (6.2)
other	3 (4.6)
Metastasis	
Present	15 (23.1)
Absent	50 (76.9)

### Cell culture and transfection

The human osteosarcoma cell lines (including MG-63,U2OS,Saos-2 and SJSA-1), the normal bone cell line hFOB, and HEK293 cell line were purchased from the American Type Culture Collection (Manassas,VA, USA). These cell lines were cultured in Dulbecco’s modified Eagle medium (DMEM; Gibco, Invitrogen Life Technologies, Carlsbad, CA, USA) supplemented with 10 % fetal bovine serum (FBS). Cells were incubated at 37 °C in 5 % CO_2_ humidity, and were passaged every 2–3 days. MiR-138 mimic (40 nM), miR-138 inhibitor (40 nM), DEC2-pcDNA3.1 (100 ng), DEC2 siRNA (100 ng) and the negative controls, including miR-Control (40 nM), pcDNA3.1 vector (100 ng), siRNA-Contrl (100 ng) were all purchased from GenePharma (Shanghai, China), and were transfected using Lipofectamine 2000 (Invitrogen Life Technologies), according to the manufacturer’s instructions. About 48 h after transfection, the transfection efficiency was assessed. The cells could be used for subsequent analysis when the transfection efficiency was above 80 %.

### Real-time quantitative reverse transcription PCR

The miRNAs were isolated from osteosarcoma tissues or cell lines using a RNeasy/miRNeasy Mini kit (Qiagen, Limburg, The Netherlands) according to the manufacturer’s instructions. Total RNA was isolated using Trizol reagent (Invitrogen). The cDNAs were synthesized using a RevertAid™ First Strand cDNA Synthesis kit (Fermentas, Vilnius, Lithuania), and real-time quantitative PCR was carried out using the SYBR-Green PCR Master Mix (Applied Biosystems, Foster City, CA, USA) on a 7900 Real-Time PCR System (Applied Biosystems). Primers used in this study were: miR-138, 5′-agctggtgttgtgaatcaggccg-3′(forward), 5′-tggtgtcgtggagtcg-3′(reverse); U6, 5′-ctcgcttcggcagcaca-3′(forward), 5′-aacgcttcacgaatttgcgt-3′(reverse); DEC2, 5′-gcctaccgtcccacagatta-3′ (forward) and 5′-tgtcgtctcgtttcatgctc-3′ (reverse); GAPDH, 5′-cgaccactttgtcaagctca-3′ (forward) and 5′-aggggtctacatggcaactg-3′ (reverse). miR-138 mRNA expression was normalized to U6 internal control, and DEC2 mRNA expression was normalized to GAPDH internal control.

### Western blot analysis

Total protein extracts were prepared using RIPA buffer with protease inhibitor Cocktail (Pierce, Rockford, IL, USA). Protein concentrations were examined using the BCA Protein Assay Kit (Pierce). Total proteins (20 μg) were separated on 10 % SDS PAGE and then transferred onto polyvinylidene fluoride membranes (Millipore, Billerica, MA, USA). After blocking with 5 % non-fat milk at 4 °C overnight, the membranes were incubated with primary antibodies against DEC2 (1:1000, mouse monoclonal anti-DEC2, Santa Cruz Biotechnology, Santa Cruz, CA, USA) or GAPDH (1:5000, mouse monoclonal anti-GAPDH, Abcam, Cambridge, MA, USA) at 4 °C overnight, followed by incubation with the secondary antibody (1:5000, rabbit anti-mouse IgG-HRP, Santa Cruz Biotechnology) for 2 h at room temperature. Protein bands were developed using the ECL western blotting kit (Pierce), and the band intensity was quantified using Image J software (National Institutes of Health, Bethesda, MD, USA).

### Dual-luciferase reporter assay

The DEC2 3′-untranslated region (3′UTR) containing the wild type or mutated miR-138 binding sequences were synthesized by Genescript (Nanjing, Jiangsu, China), and were cloned into the pmirGLO luciferase reporter vector (Promega, Madison, WI, USA). HEK293 cells were transfected with the wild type/mutant DEC2 luciferase reporter vector and miR-138 mimic/miR-Control using Lipofectamine 2000. Firefly and Renilla luciferase activities were measured using the Dual-Luciferase Reporter Assay System (Promega). Results were expressed as the firefly luciferase activity normalized to Renilla luciferase activity.

### Cell proliferation assay

Cell proliferation was determined by MTT assay. The cells were suspended after transfection, and seeded into the 96-well plates at a density of 1.5 × 10^3^ cells/well. The cells were allowed to grow for 24,48,72 and 96 h, and then 10 μl of MTT solution (0.5 mg/ml; Sigma, St.Louis, MO, USA) was added to each well and incubated at 37 °C for 4 h. 150 μl of DMSO (Sigma) was added to dissolve the formazan crystals. Cell viability was detected by measurement of the absorbance at 570 nm using a microplate reader (ELx800NB; BioTek Instruments, Inc., Winooski, VT, USA).

### Cell apoptosis assay

Forty-eight h after transfection, the cells were collected and washed with PBS, and then resuspended in 500 μl of binding buffer. 5 μl of Annexin V-FITC and 5 μl of propidium iodide (PI) (Kaiji Biological Inc., Nanjing, Jiangsu, China) were added to the samples, and incubated at room temperature for 5 min in the dark. Positive cells were analyzed by flow cytometry (FCM) (BD FACSAria; BD Biosciences, Franklin Lakes, NJ, USA).

### Transwell-Matrigel invasion assay

Transwell-Matrigel invasion assay was performed using the Transwell inserts (Corning, New York, NY, USA) coated with Matrigel (BD Biosciences, Franklin Lakes, NJ, USA). The cells were seeded to the upper chambers in serum-free medium at the density of 5 × 10^4^ cells/ml and in the amount of 2 ml/well. The lower chambers were filled with DMEM containing 10 % FBS. After incubation at 37 °C for 24 h, the non-invaded cells were wiped with a cotton swab. The invaded cells were fixed in 95 % ethanol and stained with hematoxylin. The number of invaded cells was counted under a microscope in at least five fields.

### Statistical analysis

Statistical analysis was performed on SPSS 19.0 statistical software (SPSS, Inc., Chicago, IL, USA), and all the data are presented as the means ± standard deviation. A two-tailed Student’s t-test was used for comparison of difference between 2 groups. *P* < 0.05 was considered to indicate a statistically significant difference in the study.

## Results

### Mir-138 expression was downregulated in human osteosarcoma tissues and cell lines

**Fig 1:**
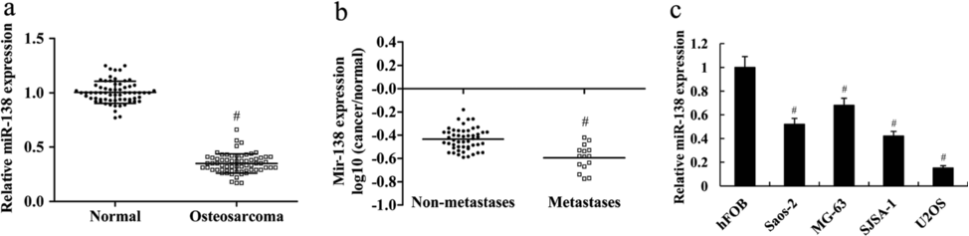
Mir-138 expression was downregulated in human osteosarcoma tissues and cell lines. **a** Fold change of miR-138 expression in human osteosarcoma tissues and the adjacent normal tissues. ^#^ *P* < 0.01 compared with the normal. **b** Relative expression of miR-138 in the osteosarcoma tissues from the patients with metastases and non-metastases. ^#^ *P* < 0.01 compared with the non-metastases tissues. **c** Fold change of miR-138 expression in human osteosarcoma cell lines (MG-63, U2OS, SJSA-1, and SAOS-2) and the normal bone cell line (hFOB). ^#^ *P* < 0.01 compared with hFOB

The expression level of miR-138 was quantified by real-time quantitative reverse transcription PCR in primary osteosarcoma tissues and osteosarcoma cell lines. Results showed that compared with the adjacent normal tissues, the expression level of miR-138 was significantly downregulated in human osteosarcoma tissues (Fig. 1a, *P* < 0.01). Moreover, miR-138 expression was significantly lower in metastatic osteosarcoma tissues than that in non-metastatic tissues (Fig. 1b, *P* < 0.01). Compared with the normal bone cell line hFOB, the expression levels of miR-138 were significantly downregulated in osteosarcoma cell lines, including MG-63, U2OS, SJSA-1, and Saos-2 (Fig. 1c, *P* < 0.01).

### Mir-138 inhibited osteosarcoma cell proliferation and invasion, and promoted cell apoptosis

**Fig 2:**
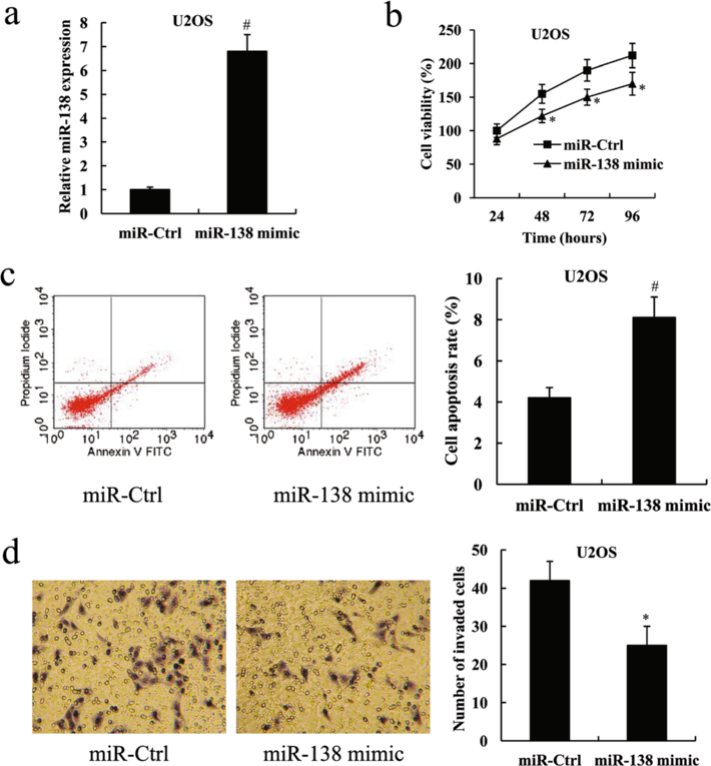
Mir-138 mimic inhibited U2OS cell proliferation and invasion, and promoted cell apoptosis. **a** Fold change of miR-138 expression in U2OS cells following transfection with the miR-138 mimic. **b** Cell viability **c** Cell apoptosis rate **d** Cell invasion of U2OS cells following transfection with the miR-138 mimic. ^*^ *P* < 0.05 and ^#^ *P* < 0.01 compared with the miR-Ctrl

**Fig 3:**
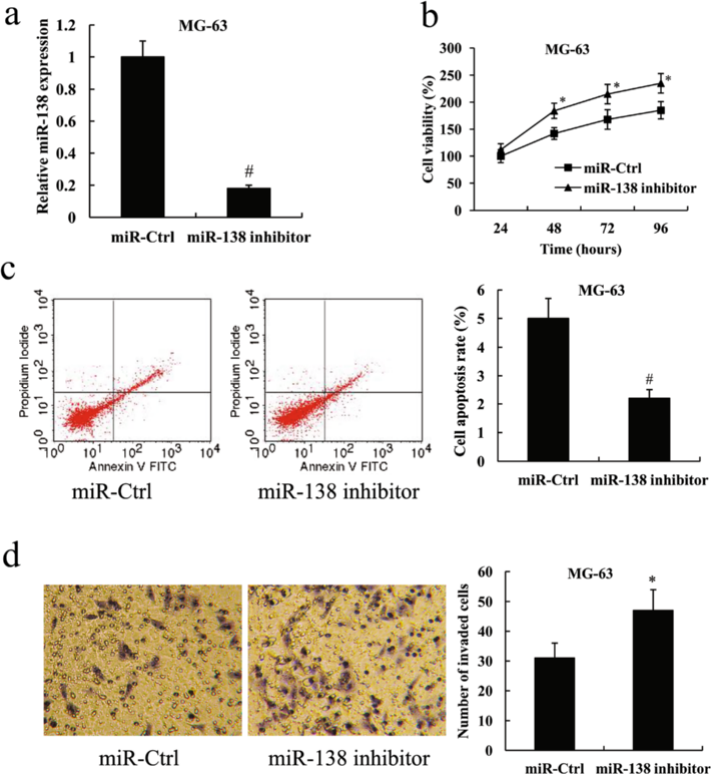
Mir-138 inhibitor promoted MG-63 cell proliferation and invasion, and inhibited cell apoptosis. **a** Fold change of miR-138 expression in MG-63 cells following transfection with the miR-138 inhibitor. **b** Cell viability **c** Cell apoptosis rate **d** Cell invasion of MG-63 cells following transfection with the miR-138 inhibitor. ^*^ *P* < 0.05 and ^#^ *P* < 0.01 compared with the miR-Ctrl

We performed gain-of-function and loss-of-function experiments to explore the role of miR-138 in human osteosarcoma. To overexpress miR-138, miR-138 mimic was transfected into the U2OS cells, which have low endogenous miR-138 expression. In addition, miR-138 inhibitor was transfected into the MG-63 cells, which exhibit high miR-138 expression, to knock down miR-138 expression. The results from real-time quantitative reverse transcription PCR analysis confirmed the ectopic overexpression of miR-138 in U2OS cells and the depletion of miR-138 expression in MG-63 cells (Figs. 2a and 3a, *P* < 0.01). 48 h after transfection, the cells were subjected to cell proliferation, apoptosis and invasion assays.

Cell proliferation was determined by MTT assay, and the results showed that miR-138 overexpression significantly suppressed cell viability in U2OS cells, whereas miR-138 knockdown significantly enhanced cell viability in MG-63 cells (Figs. 2b and 3b, *P* < 0.05 for 48,72 and 96 h).

Flow cytometry indicated that U2OS cells with ectopic overexpression of miR-138 showed a significant increase in cell apoptosis rate compared with the control. On the contrary, the knockdown of miR-138 in MG-63 cells resulted in a significantly reduced cell apoptosis rate (Figs. 2c and 3c, *P* < 0.01).

We further evaluated the effect of miR-138 on cell invasion of osteosarcoma cells. Transwell-Matrigel invasion assay demonstrated that the invasive ability of U2OS cells transfected with the miR-138 mimic was much weaker compared with the cells transfected with the miR-control (miR-Ctrl); however, the invasive ability was significantly enhanced in MG-63 cells following transfection with the miR-138 inhibitor (Figs. 2d and 3d, *P* < 0.05).

### DEC2 was a direct target of miR-138

**Fig 4:**
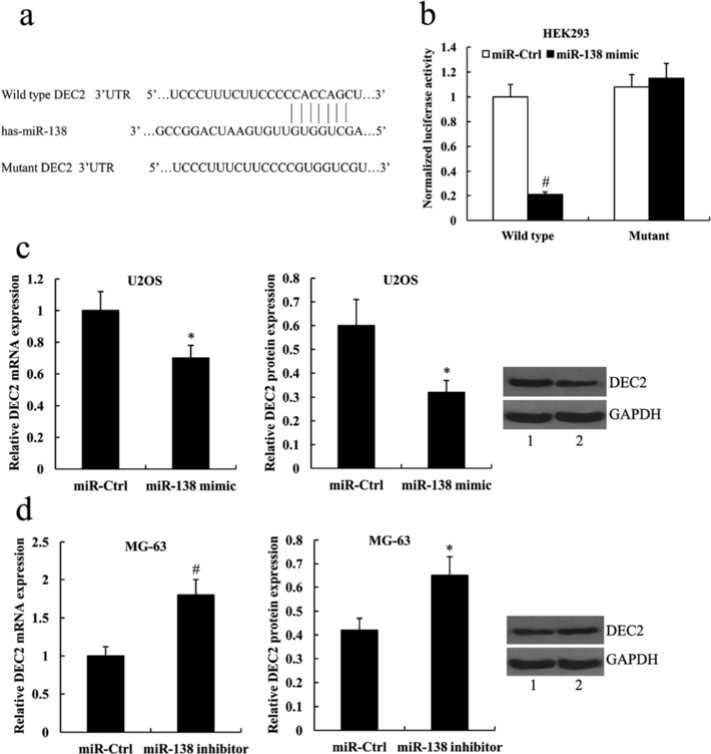
DEC2 was a direct target of miR-138. **a** The DEC2 3′UTR region containing the wild type or mutant binding site for miR-138. **b** The relative luciferase activity of DEC2 wild type or mutant 3'UTR in HEK293 cells following transfection with the miR-138 mimic. **c** Fold change of DEC2 mRNA expression and relative DEC2 protein expression in U2OS cells following transfection with the miR-138 mimic. Lane 1, miR-Ctrl; lane 2, miR-138 mimic. **d** Fold change of DEC2 mRNA expression and relative DEC2 protein expression in MG-63 cells following transfection with the miR-138 inhibitor. Lane 1, miR-Ctrl; lane 2, miR-138 inhibitor. ^*^ *P* < 0.05 and ^#^ *P* < 0.01 compared with the miR-Ctrl

DEC2 is predicted to be a potential target of miR-138 by miRanda (http://www.microrna.org/). The predicted interaction site of 7 bp of miR-138 and DEC2 3′UTR was shown in Fig. 4a. To identify whether DEC2 is a direct target of miR-138, wild type and mutant DEC2 3′UTR containing the putative target site of miR-138 were cloned into reporter plasmids respectively, and were transfected into the HEK293 cells along with the miR-138 mimic, or the control miRNA. As validated by luciferase reporter assay, the luciferase activity of wild type DEC2-3′UTR was significantly suppressed in the cells transfected with the miR-138 mimic compared with the cells transfected with the miR-Ctrl (*P* < 0.01); however, miR-138 mimic did not affect the luciferase activity of mutant DEC2-3′UTR (Fig.4b).

Furthermore, we investigated the effect of miR-138 on DEC2 expression in human osteosarcoma cell lines. We found that miR-138 mimic significantly reduced DEC2 expression at the mRNA and protein level (0.60 ± 0.11 vs.0.32 ± 0.05, *P* < 0.05) in U2OS cells. In contrast, miR-138 inhibitor significantly increased the expression of DEC2 mRNA and protein (0.42 ± 0.05 vs.0.65 ± 0.08, *P* < 0.05) in MG-63 cells (Fig. 4c,d).

### DEC2 attenuated the effects of miR-138 on osteosarcoma cells

**Fig 5:**
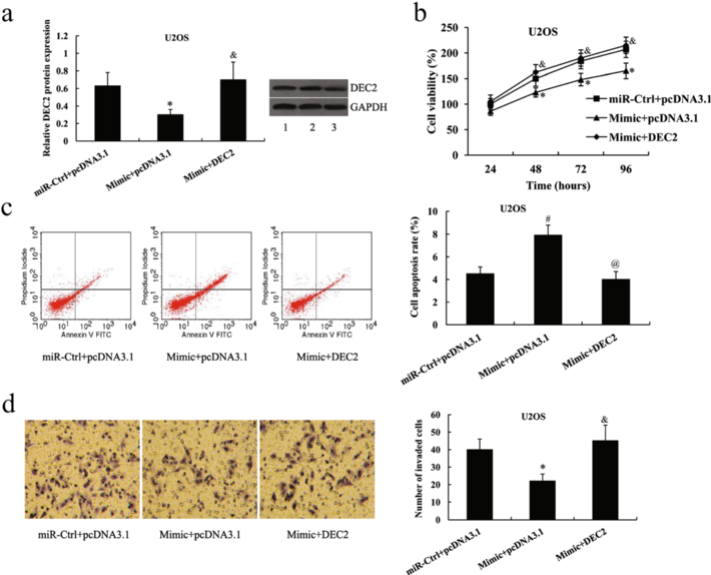
DEC2 overexpression attenuated the effects of miR-138 mimic on osteosarcoma cells. **a** Relative DEC2 protein expression in U2OS cells following transfection with the miR-138 mimic and DEC2 pcDNA3.1. Lane 1, miR-Ctrl + pcDNA3.1; lane 2, Mimic + pcDNA3.1; lane 3, Mimic + DEC2. **b** Cell viability **c** Cell apoptosis rate **d** Cell invasion of U2OS cells following transfection with the miR-138 mimic and DEC2 pcDNA3.1.^*^ *P* < 0.05 and ^#^ *P* < 0.01 compared with the miR-Ctrl + pcDNA3.1; &*P* < 0.05 and ^@^ *P* < 0.01 compared with the Mimic + pcDNA3.1

**Fig 6:**
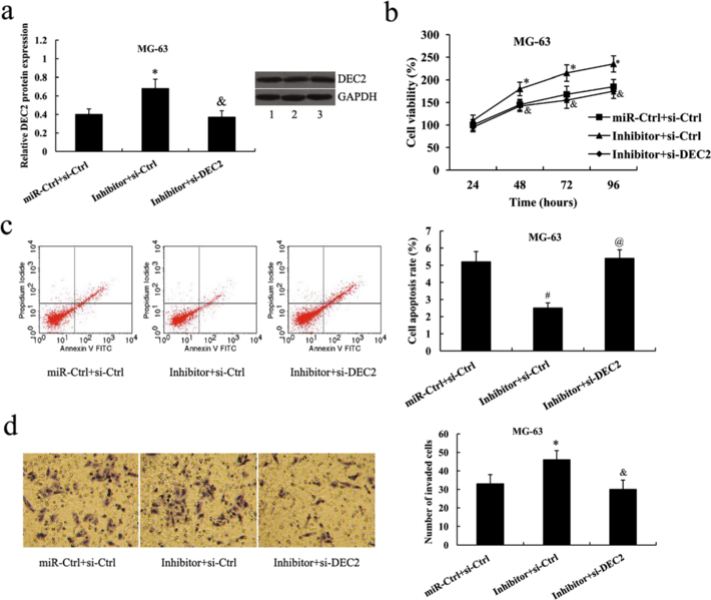
DEC2 suppression attenuated the effects of miR-138 inhibitor on osteosarcoma cells. **a** Relative DEC2 protein expression in MG-63 cells following transfection with the miR-138 inhibitor and DEC2 siRNA. Lane 1, miR-Ctrl + si-Ctrl; lane 2, Inhibitor + si-Ctrl; lane 3, Inhibitor + si-DEC2. **b** Cell viability **c** Cell apoptosis rate **d** Cell invasion of MG-63 cells following transfection with the miR-138 inhibitor and DEC2 siRNA.^*^ *P* < 0.05 and ^#^ *P* < 0.01 compared with the miR-Ctrl + si-Ctrl; &*P* < 0.05 and ^@^ *P* < 0.01 compared with the Inhibitor + si-Ctrl

To further illustrate whether miR-138 affects human osteosarcoma cell proliferation, apoptosis and invasion through DEC2, DEC2 overexpression plasmid was transfected into the U2OS cells in the presence of miR-138 mimic, whereas DEC2 siRNA was transfected into the MG-63 cells in the presence of miR-138 inhibitor. As shown in Figs. 5a and 6a,the expression of DEC2 protein was significantly increased in U2OS cells (0.30 ± 0.06 vs.0.70 ± 0.20, *P* < 0.05) but decreased in MG-63 cells (0.68 ± 0.10 vs.0.37 ± 0.07, *P* < 0.05).

Forty-eight h after transfection, cell proliferation, apoptosis and invasion assays were performed. The increased cell proliferation and invasion, and decreased cell apoptosis in U2OS cells by miR-138 mimic transfection was attenuated by the overexpression of DEC2 (proliferation, *P* < 0.05 for 48,72 and 96 h, Fig. 5b; apoptosis, *P* < 0.01, Fig. 5c; invasion, *P* < 0.05, Fig. 5d). In MG-63 cells, the effect of miR-138 inhibitor on cell proliferation, apoptosis and invasion was also reversed by DEC2 suppression (proliferation, *P* < 0.05 for 48,72 and 96 h, Fig. 6b; apoptosis, *P* < 0.01, Fig. 6c; invasion, *P* < 0.05, Fig. 6d).

## Discussion

MiR-138 has been proven to be a tumor suppressor gene in various types of tumors. Long et al. reported that downregulation of miR-138 in human colorectal cancer tissues was associated with lymph node metastasis, distant metastasis, and predicted poor prognosis. Ectopic expression of miR-138 can inhibit colorectal cancer migration and invasion in vitro and in vivo [12]. MiR-138 is also downregulated in HNSCC, and ectopically overexpressed miR-138 in HNSCC suppressed cell invasion and led to cell cycle arrest and apoptosis [17]. In ovarian cancer, miR-138 can suppress cell invasion and metastasis by targeting SRY-box 4 (SOX4) and hypoxia inducible factor 1, alpha subunit (HIF-1α) [18]. A study by Poos et al. reveals that miR-138 is significantly downregulated in proliferative active osteosarcoma cell lines, and downregulation of miR-138 results in the upregulation of its direct target genes which are involved in focal adhesion [20]. In the present study, we found that miR-138 was obviously downregulated in human osteosarcoma tissues and cell lines. Moreover, miR-138 expression was significantly lower in metastatic osteosarcoma tissues than that in non-metastatic tissues. These findings suggested that miR-138 may play important roles in the progression of osteosarcoma. Subsequently, we performed the in vitro gain-of-function and loss-of-function experiments to elucidate the role of miR-138 in osteosarcoma. We found that miR-138 inhibited cell proliferation and invasion, and promoted cell apoptosis of human osteosarcoma cells. Here, for the first time, we confirmed that miR-138 acts as a tumor suppressor in human osterosarcoma. Our report is consistent with the previous studies showing the tumor suppressor role of miR-138 in other types of tumors [12, 14, 17–19]. The further step of this study need to investigate the association between miR-138 expression and clinicopathological parameters of osteosarcoma patients.

Several target genes of miR-138 have been identified and verified in previous studies, such as vimentin, zinc finger e-box binding homeobox 2 (ZEB2), GRK-Interacting Protein 1 (GIT1) and semaphorin-4c (SEMA4C) [19, 31, 32]. In general, however, one miRNA has numerous target genes. Among the candidate target genes of miR-138, we focused on DEC2 because of its role as a regulator of cell proliferation, apoptosis, progression to malignancy, and carcinogenesis [22–26, 28, 29]. Furthermore, a recent study on the association between DEC2 and osteosarcoma showed that DEC2 contributes to the progression and metastasis of human osteosarcoma [30]. In the present study, we confirmed DEC2 as a novel direct target of miR-138 by luciferase reporter assay. Furthermore, we demonstrated that miR-138 suppresses DEC2 expression at the mRNA and protein level.

The present study revealed that miR-138 exerts its effect on osteosarcoma cells, at least partially, by downregulating the expression of DEC2. DEC2 functions as a transcriptional suppressor by binding to the E-box sequence in the promoter of various genes [33–35]. Some studies showed that DEC2 physically interacts with and promotes HIF-1α degradation, and suppresses the malignant behaviour of human breast and pancreatic cancers [23, 29]. Recently, a study by Hu et al. found that DEC2 facilitates HIF-1α stabilization and promotes HIF-1 activation in osteosarcoma, contributing to the progression and metastasis of human osteosarcoma [30]. In this study, we found DEC2 could reverse the inhibitory effect of miR-138 on osteosarcoma progression, indicating that DEC2 acts as an oncogene in osteosarcoma. This finding was consistent with the report by Hu et al. [30]. Interestingly, DEC2 have been demonstrated to suppress the progression of breast, endometrial, pancreatic and oral cancers [13, 21, 28, 29]; however, DEC2 was suggested to be an oncogene in osteosarcoma. Therefore, we speculate that the function of DEC2 in tumor cells appears to be cell type specific.

## Conclusions

MiR-138 was downregulated in human osteosarcoma tissues and cell lines. MiR-138 could inhibit osteosarcoma cell proliferation and invasion, as well as promote cell apoptosis through downregulating the expression of DEC2. This study provided new insight into the mechanisms of osteosarcoma carcinogenesis, and suggested miR-138/DEC2 as a novel therapeutic target in osteosarcoma.

## Abbreviations

DEC2: differentiated embryonic chondrocyte gene 2.
miRNA or miR: microRNA
